# Causal associations between gut microbiome and cardiovascular disease: A Mendelian randomization study

**DOI:** 10.3389/fcvm.2022.971376

**Published:** 2022-08-30

**Authors:** Yuxuan Zhang, Xinyi Zhang, Delong Chen, Jia Lu, Qinyan Gong, Jiacheng Fang, Jun Jiang

**Affiliations:** ^1^Department of Cardiology, Second Affiliated Hospital, College of Medicine, Zhejiang University, Hangzhou, China; ^2^Department of Cardiology, The First People's Hospital of Jiashan, Jiaxing, China; ^3^Cardiovascular Key Laboratory of Zhejiang Province, Hangzhou, China

**Keywords:** Mendelian randomization, gut microbiome, cardiovascular disease, causality, *Oxalobacter*, *Clostridiaceae*

## Abstract

**Background:**

Observational studies have shown gut microbiomes were associated with cardiovascular diseases (CVDs), but their roles remain controversial, and these associations have not yet been established causally.

**Methods:**

Two-sample Mendelian randomization (MR) was used to investigate whether gut microbiome had a causal effect on the risk of CVDs. To obtain comprehensive results, we performed two sets of MR analyses, one with single nucleotide polymorphisms (SNPs) that smaller than the genome-wide statistical significance threshold (5 × 10^−8^) as instrumental variables, and the other with SNPs that lower than the locus-wide significance level (1 × 10^−5^). Summary-level statistics for CVDs, including coronary artery disease (CAD), myocardial infarction, heart failure, atrial fibrillation, stroke and its subtypes were collected. The ME estimation was performed using the inverse-variance weighted and Wald ratio methods. Sensitivity analysis was performed using the weighted median, MR-Egger, leave-one-out analysis, MR pleiotropy residual sum and outlier and MR Steiger.

**Results:**

Based on the locus-wide significance level, genetically predicted genus *Oxalobacter* was positively associated with the risk of CAD (odds ratio (OR) = 1.06, 95% confidence interval (CI), 1.03 – 1.10, *P* = 1.67 × 10^−4^), family *Clostridiaceae_1* was negatively correlated with stroke risk (OR = 0.83,95% CI, 0.75–0.93, *P* = 7.76 × 10^−4^) and ischemic stroke risk (OR = 0.823,95% CI, 0.74–0.92, *P* = 4.15 × 10^−4^). There was no causal relationship between other genetically predicted gut microbiome components and CVDs risk. Based on the genome-wide statistical significance threshold, the results showed that the gut microbiome had no causal relationship with CVDs risk.

**Conclusion:**

Our findings reveal that there are beneficial or adverse causal effects of gut microbiome components on CVDs risk and provide novel insights into strategies for the prevention and management of CVDs through the gut microbiome.

## Key messages

Several studies have shown significant alternations in the structure and composition of gut microbiome in cardiovascular diseases (CVDs) patients. However, the causality between the gut microbiome and CVDs remains unclear. In this 2-sample Mendelian randomization analysis, we found that genetically predicted genus *Oxalobacter* was positively associated with coronary artery disease risk and family *Clostridiaceae_1* was associated with decrease risk of stroke and ischemic stroke.

## Introduction

Cardiovascular diseases (CVDs) remain the leading cause of mortality and morbidity in the world, though the treatment of CVDs has advanced (1). CVDs cause 17.7 million deaths (31% of global deaths) every year, a figure equivalent to a third of all deaths in US and a quarter of all deaths in Europe (2). The causes of CVDs are not fully understood through substantial progress in prevention and control (3). Therefore, it remains crucial to identify protective or causative factors for CVDs.

The human gut microbiome is a complex ecosystem that provides essential functions to its host. Recently, several studies have shown significant alternations in the structure and composition of gut microbiome in cardiovascular diseases (CVDs) patients. Emoto et al. (4) reported the changes in gut microbiome composition in patients with coronary artery disease (CAD), such as the reduced abundance of the *Bacteroidetes* and the grown abundance of the *Lactobacillales*. Zuo et al. (5) found that *Ruminococcus, Streptococcus* and Enterococcus were overgrown in patients with atrial fibrillation (AF), and *Faecalibacterium, Alistipes, Oscillibacter*, and *Bilophila* were reduced. Research has provided further evidence of a link between gut microbiome and CVDs susceptibility through direct gut microbial transplantation. Gregory et al. (6) shown that atherosclerosis susceptibility can be transmitted via transplantation of gut microbiota. However, the causal association between the gut microbiome and CVDs remains unclear, as many other factors such as age, gender and ethnicity can influence not only gut microbiome but also CVDs development, which complicating this matter. What's more, evidence from traditional epidemiological studies fails to address the confusion caused by various biases and reverse causality and is limited by small sample sizes.

In this context, Mendelian randomization (MR) provides a way to explore causality between exposures and outcomes without any potentially detrimental intervention (7). In this study, we performed a 2-sample MR study to elucidate the potential impact of genetically predicted gut microbiome on 9 CVDs: CAD, myocardial infarction (MI), AF, heart failure (HF), and stroke and its subtypes. We also performed multivariable Mendelian randomization (MVMR) to assess the potential mediating effects of blood pressure on the identified causal associations, as numbers of studies have demonstrated that blood pressure is a risk factor for CVDs (8, 9).

## Materials and methods

### Study design

**Figure 1:**
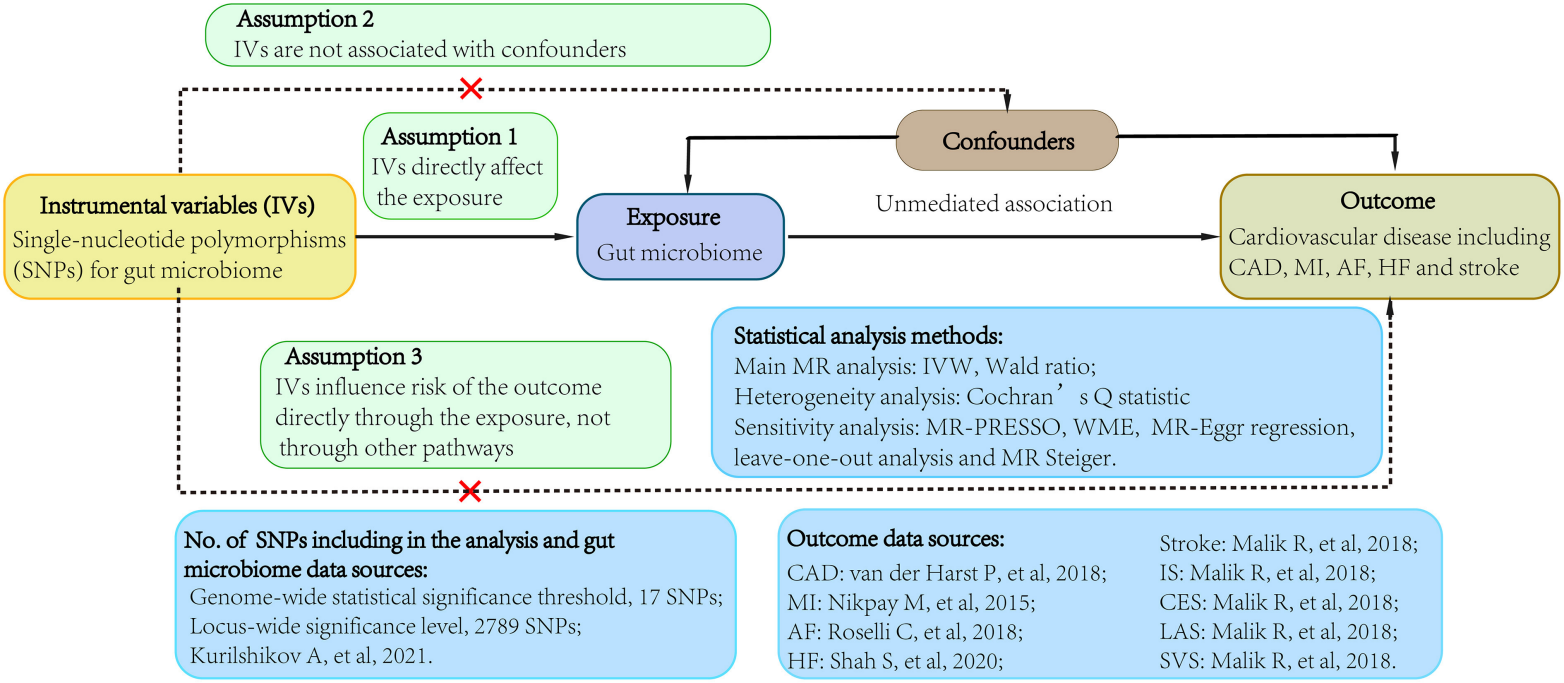
Study flow diagram. Dashed lines indicate potential pleiotropic or direct causal effects between variables that may violate MR assumptions. IV, instrumental variable; CAD, coronary artery disease; MI, myocardial infarction; HF, heart failure; AF, atrial fibrillation; IS, ischemic stroke; CES, cardioembolic stroke; LAS, large-artery atherosclerotic stroke; SVS, small-vessel stroke; IVW, multiplicative random effects inverse-variance weighted; MR, Mendelian randomization; WME, weighted-median estimator; MR-PRESSO, MR pleiotropy residual sum and outlier.

We conducted a 2-sample MR study using data obtained from the publicly available GWAS catalog to investigate the causality between gut microbiome and CVDs (http://www.ebi.ac.uk/gwas). Ethical approval and consent to participate were given in the original publications. Figure 1 shown an overview of the study design.

### Selection of genetic instrumental variables

Single nucleotide polymorphisms (SNPs) associated with the composition of human gut microbiome were selected as instrumental variables (IVs), which from a large-scale, multiethnic GWAS study involving 18,473 individuals from various countries with 122,110 loci of variation (10). To obtain more comprehensive results, this study collected two groups of SNPs (11). One group was lower than the genome-wide statistical significance threshold (5 × 10^−8^) and the other group was smaller than the locus-wide significance level (1 × 10^−5^) (11). All SNPs were required to independently (linkage disequilibrium [LD] (9), *r*^2^ ≤ 0.01) predicted human microbiome composition. Only the one with the lowest *P*-value was selected if there were SNPs with highly linkage disequilibrium. To prevent potential pleiotropy, we further searched these SNPs using PhenoScanner V2 (http://www.phenoscanner.medschl.cam.ac.uk/) to assess whether the IVs were potentially related to confounders or risk factors for CVDs (Supplementary Table 12) (12, 13). The IVs were excluded from the analysis once they were potentially related to confounders or risk factors for CVDs, such body mass index, past tobacco smoking, low density lipoprotein or other factors that have been reported (14–16).

### Outcome data sources

Summary-level data for CAD were extracted from a large-scale meta-analysis GWAS including 122,733 cases and 424,528 controls from the CARDIoGRAMplusC4D consortium and UK Biobank (17). Summary statistics for MI were also came from a large-scale GWAS meta-analysis of 48 studies from CARDIoGRAMplusC4D consortium with 43,676 cases and 128,199 controls (18). Genetic associations with AF were derived from a large-scale GWAS that comprised 55,114 cases and 482,295 controls of European ancestry (19). Summary data for HF obtained from a GWAS meta-analysis of 26 studies from HERMES consortium, including 47,309 cases and 930,014 controls of European ancestry (20). Aggregated data for stroke were came from a large-scale meta-analysis GWAS conducted by MEGASTROKE consortium, which comprised 40,585 cases and 406,111 controls of European ancestry (21). Among these cases, 34,217 patients were ischemic stroke, which was further divided into three subtypes, including 7,193 cardioembolic stroke cases, 4,373 large-artery atherosclerotic stroke cases and 5,386 small-vessel stroke cases. There was no overlap between the exposures and outcomes GWASs population.

### Instrument strength

The variance (*R*^2^) in the MR studies stands for the proportion of the variability of the exposure explained by each genetic instrument (22). Based on previously study, the *R*^2^ for the gut microbiomes was calculated as the following formula: *R*^2^ = 2 × *EAF* × (1−*EAF*) × *beta*^2^/[2 × *EAF* × (1−*EAF*) × *beta*^2^+2 × *EAF* × (1−*EAF*) × *N* × *se*^2^], where EAF means effect allele frequency, beta and se means the estimated effect and its standard error of SNP on certain gut microbiome, and N means the sample size (22). Furthermore, we used the following formula to calculate the F-statistics to evaluate the weak instrument bias: *F* = *R*^2^ × (*N*−2)/(1−*R*^2^), where *N* refers to the sample size (23).

### Statistical analysis

In this 2-sample MR, we harmonized the effect of gut microbiomes and CVD datasets, which comprised comprehensive information on SNPs, especially effect allele, standard error, beta-coefficient, *P*-value and sample size. When a specific gut microbiome-associated SNPs were missing from the outcome datasets, proxy SNPs (*r*^2^ > 0.8) were applied. SNPs without suitable proxies were excluded from the analyses. The multiplicative random effects inverse variance-weighted (IVW) method was applied for the primary MR analysis, which meta-analyzed the SNP-specific Wald estimates with the assumption of balanced pleiotropy (24). The Wald ratio method was performed when the MR estimate contained only one single SNP.

### Sensitivity analysis

To examine the existence of horizontal pleiotropy that violated the main MR assumptions, this study performed several statistical tests. Cochran Q statistic was calculated to quantify the heterogeneity in effect sizes produced from the selected genetic IVs. An MR pleiotropy residual sum and outlier (MR-PRESSO) analysis was also be applied to check and adjust for horizontal pleiotropy by removing outliers (25).In addition, an MR-Egger regression and weighted-median estimator (WME) were performed for sensitivity analyses. We estimated the deviation of the MR-Egger intercept to detect the horizontal pleiotropy, and the difference from 0 indicating potential bias in the MR estimates (26). The WME method was supplemented to generate robust and consistent estimates of the effect, even though up to 50% of the weight came from invalid IVs (27). We also applied a leave-one-out analysis to detect for any pleiotropy affected by a single SNP. Besides, the MR Steiger test was performed to evaluate the potential effect of reverse causality of CVDs on gut microbiome (28).

To consider multiple-testing correction, the significance threshold for various taxa levels was set as *P* = 0.05/n, n means the taxa size. For example, the significance threshold for locus-wide significance level group was as following: class *P* = 3.13 × 10^−3^= 0.05/16, family *P* = 1.43 × 10^−3^= 0.05/35, genus *p* = 3.82 × 10^−4^ = 0.05/131, order *P* = 2.50 × 10^−3^= 0.05/20, and phylum *P* = 5.56 × 10^−3^= 0.05/9. In MVMR, a cutoff of *P* = 0.05 was given. All these analyses were implemented using the “TwoSampleMR” package in R Version 4.1.2.

## Results

### Genetic instruments for gut microbiomes

After removing SNPs that had LD effects and possibly related to confounders or risk factors for CVD, a total of 2,789 (*P* < 1 × 10^−5^) and 17 (*P* < 5 × 10^−8^) SNPs were selected as IVs (Supplementary Table 1). These SNPs were categorized according to five biological categories, including phylum, class, order, family and genus. For instance, a total of 2,672 SNPs is associated with CAD in locus wide significance level which then categorized into 9 phyla (115SNPs), 16 classes (216 SNPs), 20 orders (265 SNPs), 35 families (470 SNPs), 131 genera (1606 SNPs), and A total of 14 SNPs is associated with CAD in genome-wide statistical significance threshold. The main information of SNPs was collected systematically for further analysis, including effect allele, other allele, beta-coefficient, standard error, *P*-value and EAF.

### Locus-wide significance level

#### Causal effects of gut microbiomes on CVDs

We found evidence that genus *Oxalobacter* was positively related to CAD risk (odds ratio (OR) = 1.06, 95% confidence interval (CI), 1.03 – 1.10, *P* = 1.67 × 10^−4^) but were not associated with the other 8 CVDs. We observed that family *Clostridiaceae_1* was associated with deceased risk of stroke (OR = 0.83, 95% CI, 0.75–0.93, *P* = 7.76 × 10^−4^) and ischemic stroke (OR = 0.82, 95% CI, 0.74–0.92, *P* = 4.15 × 10^−4^), whereas the associations with the other CVDs were not significant (Table 1). In addition, no causal relationship was found between other genetically determined gut microbiome components and CVDs risk.

**Table 1:** MR results of causal links between gut microbiome and CVDs risk (*P* < 1 × 10-5).

**Outcome**	**Classification**		**N** **snp**	**Methods**	**Beta**	**SE**	**OR (95% CI)**	***P*-value**	**Horizontal pleiotropy**			**Heterogeneity**		**MR-PRESSO**
									**Egger intercept**	**SE**	***P*-value**	**Cochran's Q**	***P*-value**	***P*-value**
CAD	Genus	Oxalobacter	12	MR Egger	0.156	0.070	1.17(1.02–1.34)	0.051	−0.014	0.010	0.193	3.877	0.953	0.896
				IVW	0.061	0.016	1.06(1.03–1.10)	1.67 × 10^−4^						
				Weighted median	0.066	0.022	1.07(1.03–1.12)	0.003						
				Weighted mode	0.067	0.035	1.07(0.99–1.14)	0.081						
Stroke	Family	Clostridiaceae 1	11	MR Egger	−0.352	0.180	0.70(0.49–1.00)	0.083	0.012	0.013	0.358	14.289	0.112	0.145
				IVW	−0.186	0.055	0.83(0.75–0.93)	7.76 × 10^−4^						
				Weighted median	−0.180	0.063	0.84(0.74–0.95)	0.004						
				Weighted mode	−0.200	0.108	0.82(0.66–1.01)	0.092						
Ischemic stroke	Family	Clostridiaceae 1	11	MR Egger	−0.328	0.176	0.72(0.51–1.02)	0.095	0.010	0.012	0.449	5.405	0.798	0.851
				IVW	−0.196	0.055	0.82(0.74–0.92)	4.15 × 10^−4^						
				Weighted median	−0.195	0.076	0.82(0.71–0.96)	0.010						
				Weighted mode	−0.183	0.107	0.83(0.68–1.03)	0.117						

SNP, single nucleotide polymorphism; OR, odds ratio; MR-PRESSO, Mendelian randomization pleiotropy residual sum and outlier; CAD, coronary artery disease; IVW, multiplicative random effects inverse-variance weighted.

We then performed MVMR to adjust for CVD related traits to reassess the causality observed in our primary analysis and to explore the potential mediators (Supplementary Tables 10, 11). The causal association between genus *Oxalobacter* and CAD attenuated to null after adjusting for systolic blood pressure (SBP, OR = 1.01, 95% CI, 0.97–1.05, *P* = 0.617), diastolic blood pressure (DBP, OR = 1.00,95% CI, 0.96–1.04, *P* = 0.922), or both (OR = 1.04,95% CI, 0.99–1.09, *P* = 0.119). The causal relationship between family *Clostridiaceae_1* and stroke also weakened to null after adjusting for SBP (OR = 1.03, 95% CI, 0.97–1.09, *P* = 0.319), DBP (OR = 1.02, 95% CI, 0.96–1.08, *P* = 0.480), but causality remained when adjusted for both SBP and DBP (OR = 1.07, 95% CI, 1.00–1.13, *P* = 0.045). The causality between family *Clostridiaceae_1* and ischemic stroke also weakened to null after adjusting for SBP (OR = 1.01, 95% CI, 0.95–1.08, *P* =0.670), DBP (OR = 1.02, 95% CI, 0.95–1.09, *P* = 0.525), or both (OR = 1.05, 95% CI, 0.98–1.12, *P* = 0.205). These results suggest that gut microbiome might affect CVDs risk through blood pressure.

#### Sensitivity analyses

In the Cochran's Q statistic, heterogeneity was detected in several diseases. After using the random effects model to estimate IVW, these results did not change significantly (Supplementary Tables 3, 6). The MR-Egger regression results showed that there was no horizontal pleiotropy between the genus *Oxalobacter* and CAD (*P* = 0.896), family *Clostridiaceae_1* and stroke (*P* = 0.145), family *Clostridiaceae_1* and ischemic stroke (*P* = 0.851). The MR-PRESSO analysis showed that there are no outliers in the analysis of genus *Oxalobacter*, family *Clostridiaceae_1*. The results obtained by WME method were consistent with those achieved with the IVW method (Table 1). There was no significant change in the risk estimations for genetically predicted in leave-one-out analysis, proving that the causal relationship was not driven by specific SNPs (Supplementary Figures 1–3). There were no weak instrumental variables bias as the *F* statistics of the SNPs were all >10 (Table 2; Supplementary Table 1). The MR Steiger test indicated that there was no reverse causality (Supplementary Table 5).

**Table 2:** Characteristics of SNPs used for gut microbiome in MR analyses (*P* < 1 × 10^−5^).

**Bacterial traits**	**SNP**	**EA**	**OA**	**EAF**	**Beta**	**SE**	***P*-value**	**Sample size**	***R* ^2^**	***F* statistic**
Family Clostridiaceae1	rs56188186	A	G	0.076	0.097	0.022	8.24E-06	13,218	0.001	19.801
	rs12490337	C	G	0.246	−0.062	0.014	6.91E-06	14,253	0.001	20.297
	rs62397761	A	G	0.286	0.062	0.014	9.08E-06	14,248	0.001	20.440
	rs2817172	C	T	0.413	0.056	0.012	5.27E-06	13,789	0.001	20.665
	rs10875374	C	T	0.448	−0.054	0.012	8.10E-06	14,253	0.001	20.194
	rs881532	A	G	0.523	−0.053	0.012	7.90E-06	14,250	0.001	20.058
	rs12186080	G	A	0.839	0.075	0.016	5.34E-06	14,244	0.001	21.209
	rs550843	T	C	0.851	−0.073	0.017	7.09E-06	14,234	0.001	19.040
	rs12341505	G	A	0.889	0.081	0.018	4.54E-06	14,250	0.001	20.624
	rs4723021	T	C	0.942	−0.106	0.024	7.42E-06	13,682	0.001	19.328
	rs2795528	G	A	0.951	−0.181	0.039	3.81E-06	6,411	0.003	21.425
Genus. Oxalobacter	rs12002250	A	C	0.060	0.217	0.047	1.42E-06	4,297	0.005	21.669
	rs36057338	G	T	0.076	0.208	0.042	8.80E-07	4,244	0.006	24.312
	rs11108500	A	G	0.077	−0.199	0.043	3.74E-06	4,303	0.005	21.698
	rs1569853	T	C	0.138	−0.138	0.030	3.65E-06	4,492	0.005	21.607
	rs6000536	C	T	0.211	−0.131	0.025	2.06E-07	4,654	0.006	26.626
	rs736744	C	T	0.585	0.118	0.021	2.57E-08	4655	0.007	31.122
	rs6071435	T	A	0.636	−0.106	0.021	1.07E-06	4,635	0.005	24.098
	rs4428215	G	A	0.740	0.130	0.024	7.51E-08	4,655	0.006	28.918
	rs10464997	G	A	0.847	0.138	0.029	3.30E-06	4,650	0.005	21.805
	rs6993398	G	A	0.847	0.127	0.028	7.13E-06	4,656	0.004	20.804
	rs3862635	C	T	0.922	−0.172	0.039	9.19E-06	4,469	0.004	19.078
	rs111966731	T	C	0.928	0.213	0.047	7.30E-06	3,931	0.005	20.409

SNP, single nucleotide polymorphism; EA, effect allele; OA, other allele; SE, standard error.

### Genome-wide statistical significance threshold

When the MR analysis on gut microbiome was performed as an entire, the results of IVW, MR Egger, WME and weighted mode showed that gut microbiome was not related with any CVD risk (Supplementary Table 7). The Cochrane Q statistics results showed no significant heterogeneity except for HF (*P* = 0.049) (Supplementary Table 7). In addition, the *F* statistics of all SNPs were >10. After using the random effects model to estimate IVW, the result for HF did not change significantly (Supplementary Table 7). There was no evidence of horizontal pleiotropy between IVs and CVDs as shown by MR-Egger regression analysis (Supplementary Table 7). The results of gut microbiome classification also indicated that no causal relationship between gut microbiome and CVDs (Supplementary Table 8). Heterogeneity and horizontal pleiotropy were failed to be examined due to the limited number of included SNPs.

## Discussion

In this study, we used MR to investigate the potential causal relationship between gut microbiome and 9 CVDs. Our findings indicate that genetically predicted level of genus *Oxalobacter* might be the risk factor for CAD, and family *Clostridiaceae_1* was related to a reduced risk of stroke and ischemic stroke, probably acting through blood pressure. We failed to find evidence to support a causal association between other gut microbiome and CVDs in both locus-wide significance level and genome-wide statistical significance threshold.

A compelling finding of this study is that genus *Oxalobacter* might increase the risk of CAD. The type species of genus *Oxalobacter* is *Oxalobacter formigenes*, which has been widely studies in nephrolithiasis (29). *Oxalobacter* was thought to prevent calcium nephrolithiasis through two different mechanisms: degradation of oxalate in the gut lumen with reduction of mucosal absorption and promotion of endogenous oxalate secretion by the gut mucosa (30). However, studies on the role of *Oxalobacter* in CAD are limited. Emoto et al. (4). analyzed the gut microbiota composition in CAD patients for the first time but did not find a link between the genus *Oxalobacter* and CAD. Recently, Zheng and her colleagues (31) found that 28 genera, including *Oxalobacter*, were significantly increased in CAD patients, which was consistent with our findings. It is a pity that Zheng et al. did not conduct further research and discussion on this finding. Thus, it is necessary to further study the possible role of *Oxalobacter* in CAD development.

Although many studies have examined changes in *Clostridiaceae* in stroke patients, the relationship between them remains unclear. Previous studies have found an overabundance of *Clostridial* species in post-stroke mice, which as a part of stroke-induced shift in microbiological composition (32). On the contrast, Lee et al. (33) found that Clostridiaceae were enrich in microbiota of young stroke mice compared to aged and associated with improved outcomes. In human, Xia et al. (34) found that stroke patients had decreased abundance of *Clostridiaceae* compared to health control, which was accord with our findings. They also found that decreased abundance of *Clostridiaceae* was strongly associated with more severe brain injury and a greater likelihood of unfavorable outcomes. It should be noted that the results of this study suggested that *Clostridiaceae* was related to both stroke and ischemic stroke, but ischemic stroke account for 85% of all stroke cases. This suggests the causality of *Clostridiaceae* and stroke may be due to its causal relationship with ischemic stroke to some extent.

The mechanisms of the neuroprotective effects of *Clostridiaceae* on stroke are not fully understood. One possibility is the protective effect of *Clostridiaceae*-driven short-chain fatty acids (SCFAs) on ischemic stroke. Studies have demonstrated that higher risk of stroke was correlated with lessened levels of butyrate-producing bacteria in the Gut microbiome (35). Sun et al. (36) observed that *Clostridium butyricum*, the type species of *Clostridiaceae*, has a neuroprotective effect against cerebral ischemia/reperfusion injury mice, and this neuroprotective effect may be involved to its ability to reverse the decrease of butyric acid content in the brain. Furthermore, researchers have shown that poor stroke outcome in older mice can be reversed by poststroke bacteriotherapy supplemented with 4 SCFA-producers, including *Clostridium symbiosum* (33).

In the initial studies, trimethylamine N-oxide (TMAO), a metabolite formed after diet, has attracted extensive attention as a potential causal link between gut microbiome and CVDs. Wang et al. (37) first identified TMAO as a gut microbiota-derived factor that has been shown to predict risk for CVDs. Studies have observed that high TMAO levels were positively associated with the risk of major cardiovascular events (38).A meta-analysis of date from 19 studies shown that participants with higher levels of TMAO had a 62% increased risk for the development of cardiovascular events (39). However, researchers failed to demonstrate a significant association of genetically predicted higher levels of TMAO and its predecessor with cardiometabolic disease through MR (40).

Our study has several advantages. The main advantage is the MR design, which enables us to estimate the causality between gut microbiome and 9 CVDs without disturbance from residual confounding or reverse causal relationship. We strictly screen the related SNPs using Plink clumping and Phenoscanner before the MR analysis. As far as we know, this is the first MR analysis on this topic.

There are also several limitations in this study. First, the available data we used were not individual-level statistics, so it might lead to inevitable biases in our results. Second, due to the lack of demographic data in the original research, we were unable to conduct the subgroup analysis, such as gender-specific causal association between gut microbiome and the risk of CVDs. Third, gut microbiome gene regulation can be greatly influenced by epigenetic phenomena (e.g., methylation) and developmental compensation mechanism (41), which may also influence the association between gut microbiome and CVDs, but such effects cannot be assessed because these are inherent defects of MR.

In conclusion, our MR study supports that there are beneficial or adverse causal effects of gut microbiome components on CVDs risk. We find suggestive evidence that genus *Oxalobacter* are causally association with higher risk of CAD and family *Clostridiaceae_1* are causally related to lower risk of stroke and ischemic stroke. Our findings provide novel insights into strategies for the prevention and management of CVDs through the gut microbiome.

## Data availability statement

The original contributions presented in the study are included in the article/Supplementary materials, further inquiries can be directed to the corresponding author.

## Ethics statement

Ethical approval was not provided for this study on human participants because ethical approval and consent to participate were carried out in the original publications. Written informed consent was not provided because ethical approval and consent to participate were carried out in the original publications.

## Author contributions

YZ, DC, JL, and JJ contributed to data analysis. YZ, XZ, DC, QG, and JF structured the manuscript giving contributed to table, figures, and text editing. JJ and YZ revisited the article implementing the final manuscript form. All authors contributed to the article and approved the submitted version.

## Funding

JJ was supported by grant from the National Natural Science Foundation of China (No. 82170332), Primary Research and Development Plan of Zhejiang Province (No. 2020C03016), and Chinese Cardiovascular Association-V.G fund (No. 2017-CCA-VG-006). XZ was supported by grant from the National Natural Science Foundation for young scientists of China (No. 82100346).

## Conflict of interest

The authors declare that the research was conducted in the absence of any commercial or financial relationships that could be construed as a potential conflict of interest.

## Publisher's note

All claims expressed in this article are solely those of the authors and do not necessarily represent those of their affiliated organizations, or those of the publisher, the editors and the reviewers. Any product that may be evaluated in this article, or claim that may be made by its manufacturer, is not guaranteed or endorsed by the publisher.
