# Supplementary figures and images for: Causal associations between gut microbiome and cardiovascular disease: A Mendelian randomization study
Source: Front Cardiovasc Med. 2022 Aug 30;9:971376. doi: 10.3389/fcvm.2022.971376 (PMC9470126; doi:10.3389/fcvm.2022.971376)

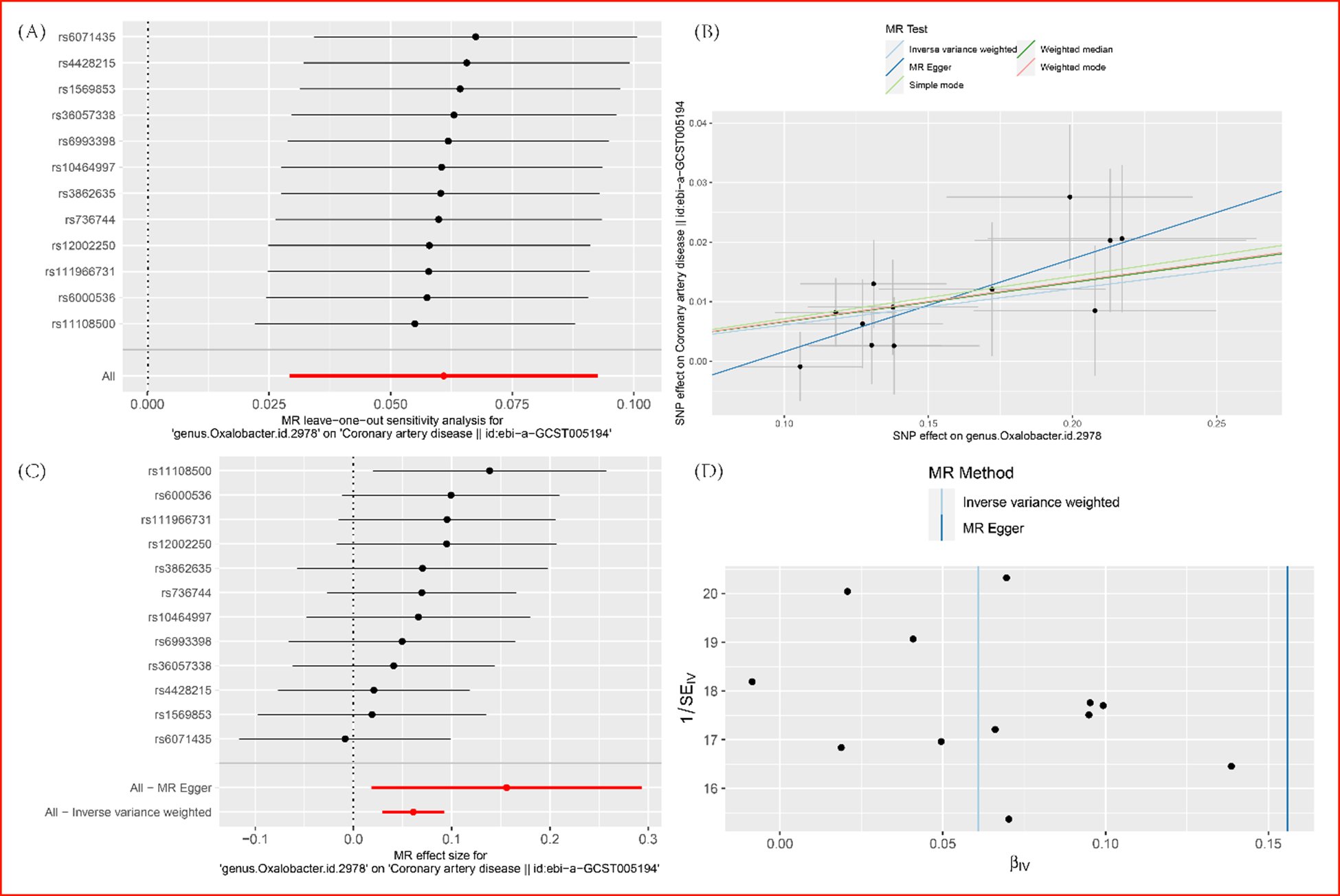

Supplement: Supplementary file 2 [file Image_1.TIF]

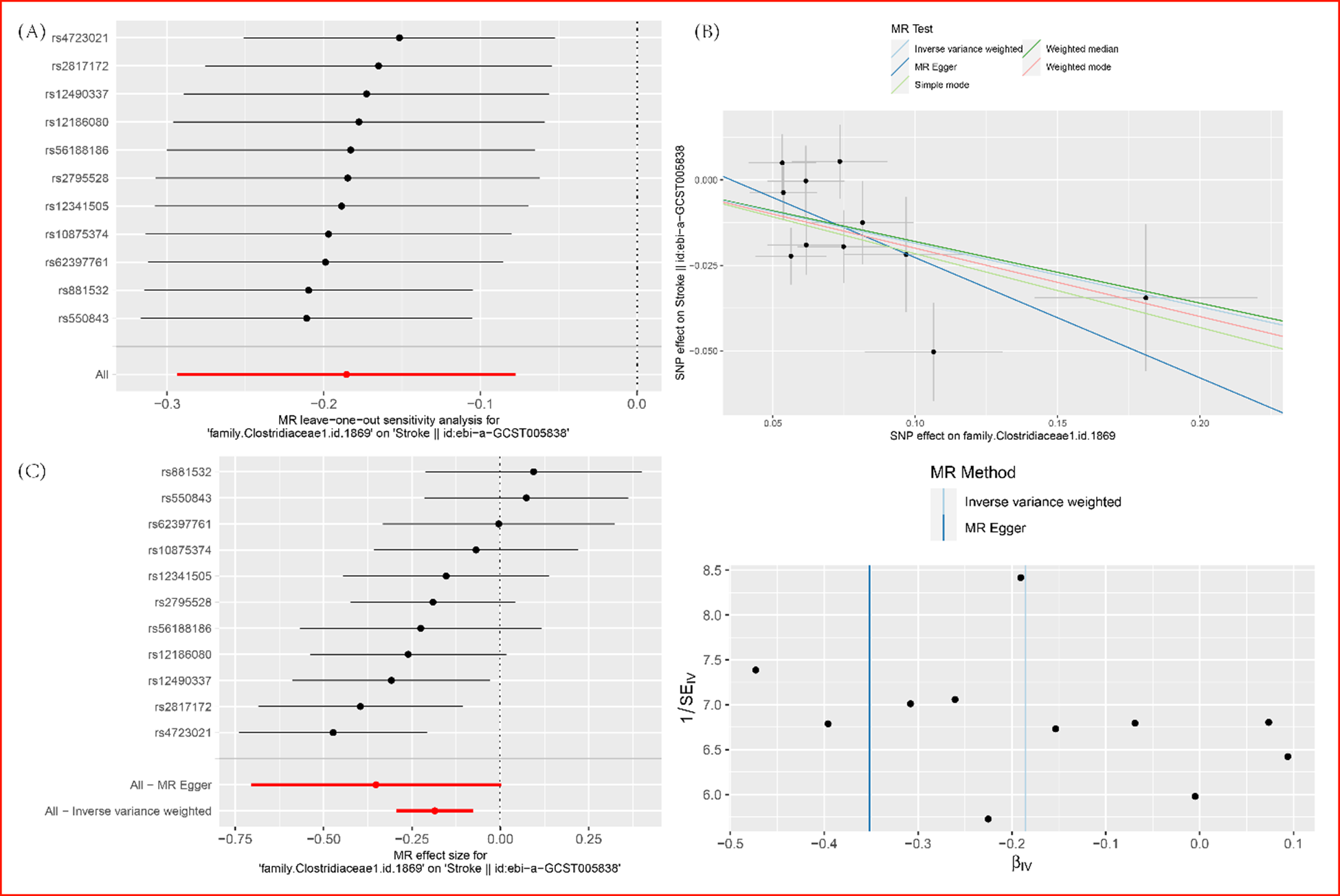

Supplement: Supplementary file 3 [file Image_2.TIF]

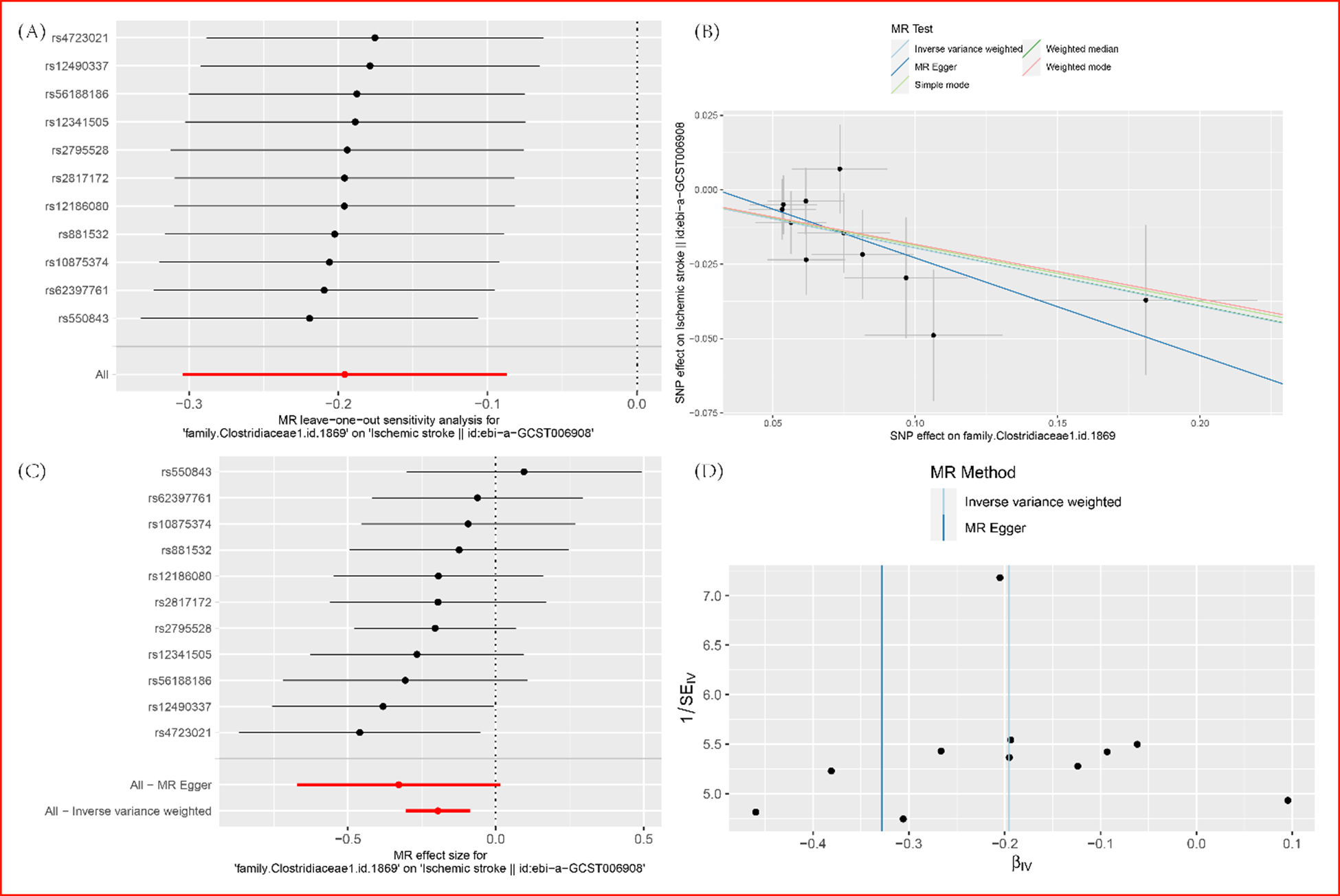

Supplement: Supplementary file 4 [file Image_3.TIF]
